# High maternal mortality in rural south-west Ethiopia: estimate by using the sisterhood method

**DOI:** 10.1186/1471-2393-12-136

**Published:** 2012-11-23

**Authors:** Yaliso Yaya, Bernt Lindtjørn

**Affiliations:** 1 Centre for International Health, University of Bergen, Bergen, Norway; 2 Arba Minch College of Health Science, Arba Minch, Ethiopia

**Keywords:** High maternal mortality, Maternal mortality, Sisterhood method, Bonke, Gamo Gofa, Southwest Ethiopia, Ethiopia, Sub-Saharan Africa

## Abstract

**Background:**

Estimation of maternal mortality is difficult in developing countries without complete vital registration. The indirect sisterhood method represents an alternative in places where there is high fertility and mortality rates. The objective of the current study was to estimate maternal mortality indices using the sisterhood method in a rural district in south-west Ethiopia.

**Method:**

We interviewed 8,870 adults, 15–49 years age, in 15 randomly selected rural villages of Bonke in Gamo Gofa. By constructing a retrospective cohort of women of reproductive age, we obtained sister units of risk exposure to maternal mortality, and calculated the lifetime risk of maternal mortality. Based on the total fertility for the rural Ethiopian population, the maternal mortality ratio was approximated.

**Results:**

We analyzed 8503 of 8870 (96%) respondents (5262 [62%] men and 3241 ([38%] women). The 8503 respondents reported 22,473 sisters (average = 2.6 sisters for each respondent) who survived to reproductive age. Of the 2552 (11.4%) sisters who had died, 819 (32%) occurred during pregnancy and childbirth. This provided a lifetime risk of 10.2% from pregnancy and childbirth with a corresponding maternal mortality ratio of 1667 (95% CI: 1564–1769) per 100,000 live births. The time period for this estimate was in 1998. Separate analysis for male and female respondents provided similar estimates.

**Conclusion:**

The impoverished rural area of Gamo Gofa had very high maternal mortality in 1998. This highlights the need for strengthening emergency obstetric care for the Bonke population and similar rural populations in Ethiopia.

## Background

Maternal mortality is defined as the death of a woman during pregnancy or within 42 days after termination of pregnancy from any cause related to or aggravated by the pregnancy or management of the pregnancy [1]. Maternal mortality is particularly high in developing countries [2], where 98% of the yearly 500,000 maternal deaths occur [3,4]. Of the 20 countries with the highest maternal mortalities in the world, 17 are in Africa. The Millennium Development Goals aim to reduce maternal deaths by 75% by 2015 from the 1990 baseline (MDG-5) [5]. The indicator chosen to measure the progress is the maternal mortality ratio (MMR; number of maternal deaths per 100,000 live births). Unfortunately, the progress in many sub-Saharan African countries has been slow or non-existent [6].

Ethiopia is one of the six countries where > 50% of the total maternal deaths worldwide occur; the other countries are the Democratic Republic of Congo, Nigeria, India, Pakistan, and Afghanistan [6]. Since 1990, Ethiopia has reduced child mortality [7]. There are also reports of reductions in MMR, but not statistically significant. The MMR for Ethiopia was 1061 (665–1639) in 1980, 968 (600–1507) in 1990, 937 (554–1537) in 2000, and 590 (358–932) in 2008 [6]; however, these estimates have wide and overlapping confidence intervals that highlight the difficulty in detecting real changes. It was as recent as 2008 that the upper uncertainty limit of the MMR decreased to < 1000. Also, there are discrepancies between estimates from different sources and methods. For example, the MMR was 590 (358–932) for 2008 according to the Institute for Health Metrics and Evaluation by Hogan et al. [6], while the UN agencies and The World Bank estimated the MMR to be 470 (270–790) [8]. Estimates of the MMR from community-based studies also vary; specifically, for 1982/83 the MMR was 566 in Addis Ababa [9], 570 (420–720) for Illubabor in western Ethiopia in 1991 [10], and between 440 (314–598) and 665 (558–785) by surveillance and sisterhood method respectively for Butajira in south central Ethiopia in 1996 [11]. These surveys showed lower estimates than the mathematically-modelled estimates for the country.

African countries, unlike developed nations, lack reliable vital registrations to provide good MMR estimates. Developed countries use birth registries and link such registries to causes of death registries, which are the gold standard by which maternal mortality is estimated. An alternative source of information includes health service data, which depends on reports of health institutions; however, the health service reports in developing countries are often biased, as only few people use these services. Also, information gathered through health services is incomplete. It is thus difficult to estimate the accurate MMRs based on institutional data [12]. Therefore, developing countries with limited heath service coverage attempt to include maternal mortality-related questions in household surveys, such as the Demographic and Health Survey (DHS). Although these surveys have contributed important information for monitoring interventions, the surveys are expensive and do not provide the regional and local estimates which are needed to improve health services.

For countries with high maternal mortality and fertility rates, Graham and colleagues [13] developed an indirect sisterhood method for calculating maternal mortality indices. This method is widely used in Africa and Asia to provide community-based maternal mortality estimates [13-15]. Unfortunately, there are no such reports from south Ethiopia. Our study aimed to determine the lifetime risk of death of women from pregnancy-related causes and to calculate the MMR in a rural area in Gamo Gofa.

## Methods

### The setting

We conducted this study in 15 of 30 randomly selected rural kebeles (lowest administrative units) in the Bonke woreda (district) of the Gamo Gofa zone in south-west Ethiopia. Bonke is one of 15 woredas in the Gamo Gofa zone and had a population of 173,240 in 2010 [16]. The woreda consists of 31 kebeles; 1 of these kebeles is a town. Geresse, the administrative centre of Bonke, is 618 km from Addis Ababa and 68 km from the zonal town, Arba Minch. However, greater than two-thirds of the people in Bonke live in highlands, which are far from roads. The only road to the woreda is the road from Arba Minch to Kamba. The road is often interrupted because of overflowing rivers during the rainy season and most of the population lives in remote villages far from the road.

The district is divided into the cold and mountainous highlands, and hot lowlands with malaria endemic to the lowland area. Healthcare is provided by a health centre at the town, and three other rural health centres. There are no medical doctors working in the district, and the health institutions are staffed by a few health officers and nurses. In the woreda, there is no access to comprehensive emergency obstetric care providing caesarean deliveries and blood transfusions. There are villages that are as far as a 14-h walk (approximately 72 km) from a road and a 20-h walk (100 km) from the nearest comprehensive emergency obstetric care at Arba Minch Hospital.

We conducted this study as part of an intervention project to reduce maternal mortality in Gamo Gofa. The work also included studies on the estimation of maternal mortality through a community-based birth registry, a retrospective 5-year recall period household survey, and a health facilities obstetric care quality study.

### The sisterhood method

In the sisterhood method, adult men and women report the proportion of their adult sisters (born to the same mother) dying during pregnancy, childbirth, or within 6 weeks following pregnancy [17]. The main objective of this method is to create a retrospective cohort of women at risk of pregnancy-related death, and to estimate the lifetime risk (LTR; the chance of a woman dying from pregnancy-related causes during her entire reproductive period). Then, the LTR is translated into the more conventional MMR.

The MMR estimate obtained through the indirect sisterhood method using respondents 15–49 years of age refers to events approximately 10–12 years before the collection of data. The time of estimation for the MMR extends up to 35 years from the time of data collection, when the respondents are older (if included, > 50 years of age). Therefore, the information obtained from such surveys is used as a quick reference of past mortality rather than of recent events. This method is not recommended for overseeing the trend over the long period of maternal mortality or for geographic comparisons [18].

To translate the lifetime risk into the MMR, the method recommends that the total fertility rate (TFR; the average number of children that would be born to a woman over her lifetime) should be ≥ 5. In 2000, the TFR for the rural Ethiopian population was 6.4 [19]. Because this rural area has a high illiteracy rate, and is a densely-populated, subsistent-farming community, we assumed the population to have similar fertility with other rural areas in Ethiopia. Therefore we used a TFR of 6.4 in the current study.

### The data collection

We recruited data collectors who had completed the 12th grade, lived in the area, and were familiar with the local language and culture. Five diploma graduates who also had a thorough knowledge of the culture and language of the area supervised the data collectors. Each enumerator was trained for 2 days. The training included pre-test field interviews, translation of the questions, and understanding the different interpretations of the questions by the respondents.

We asked men and women 15 – 49 years of age the following standard questions using the sisterhood method [17]: 

1. How many sisters (born to the same mother) have you had who survived to reproductive age (15 years of age)?

2. How many sisters who reached reproductive age (15 years of age) are alive now?

3. How many sisters died?

4. How many sisters died during pregnancy, childbirth, or 6 weeks after delivery or termination of pregnancy

In addition, we collected data on the age, gender, and education of each respondent. Fifteen years of age was considered the common age at which women are expected to undergo menarche. Therefore, we used 15 years as the proxy age for reaching reproductive age with additional probing of a reproductive age phrase itself. Data collectors were carefully trained not to include the responding woman in the reported number of sisters born to her mother.

The questions were translated to Amharic (Ethiopian official state language), and the enumerators administered Amharic using the local Gamotho language. The enumerators visited each household in the selected communities that had at least one pregnancy during the 5 years prior to the study. The enumerators asked the four questions (*vide supra*) to the husband and wife, and to the children, if any, who were 15–49 years of age.

Other extended adult family members in the household were also interviewed. If an adult person was not present during the first visit, the data collectors re-visited the household the following morning.

### Sample size and sampling technique

The sample size recommended by Graham and colleagues was 3000–6000 adult respondents [17]. A more precise recommendation of the sample size estimation, which considers the margin of error, confidence level, power of the estimate, and the required number of maternal deaths of sisters, suggests a more detailed sample size determination [20]. The formula which calculates the number of maternal deaths required for reporting by respondents was determined as follows: r ≥ [Zα/2]^2^ * [100÷% ME]^2^, where r is the number of sister deaths due to maternal causes that were required, Zα/2 is the standard normal deviate at a two-sided confidence level of 100[1-α], and the% ME is the percentage margin of error tolerated by the investigators.

We used a tolerable margin of error of 10%, and an α value of 5% (two-sided 95% CI). From the formula we calculated [1.96]^2^ * [100/10]^2^ = 384 sister deaths due to pregnancy, childbirth, or 6 weeks after the pregnancy terminated. Hanely and colleagues [20] have suggested that with 80% statistical power for a community with a MMR > 750 per 100,000 live births, a report of ≥ 384 maternal deaths is expected from interviewing 8000 adult siblings. In 2000, the MMR estimate was 937 for Ethiopia [6]. To account for non-responses and missed information, we decided to interview 9000 respondents.

We grouped the 30 kebeles of Bonke Woreda into three climatic zones (hot, temperate, and cold). To ensure fair representation of all three climatic conditions, we selected one-half of the kebeles in each climatic zone using a lottery method. Thus, we selected 8 of 16 Dega (cold weather), 4 of 8 Woinadega (moderate temperature), and 3 of 6 Kolla (hot temperature) kebeles. Then, the 9000 respondents were distributed to the study kebeles proportionate to the population size.

### Data analysis

SPSS 16 (SPSS, Inc., Chicago, IL, USA) was used for data entry and analysis [21]. We used an inflation adjustment to determine the final number of surviving adult sisters for the younger respondents (15–24 years of age. This was done by multiplying the number of respondents in the young age groups by the average number of sisters among the older respondents (25–49 years of age), which was 2.65 in this data. For example, 2.65* 2443= 6471 adjusted sisters for the 15–19 year old respondents [17]. This factor was used with the assumption that the younger respondents had sisters who had yet to reach reproductive age.

Using standard adjustment factors [17], we adjusted for the expected proportion of sisters that would have finished their reproductive age for respondents in each age category. Thus, 90% of the sisters of respondents 45–49 years of age are expected to have passed through their reproductive life, but only 10.7% of the sisters of 15–19 year old respondents. The adjustment was implemented so as to determine the number of sister units exposed to maternal death.

This retrospective cohort analysis provided 8,068 sister units exposed to the risk of maternal death that served as the denominator for calculating the lifetime risk of maternal death.

The lifetime risk (Q) of maternal death was calculated by Q=r/ β, where r is the number of maternal deaths and β is the sister units exposed to the risk of maternal death. We calculated the MMR as MMR =1-(P) ^1/TFR^, where P is the probability of surviving, which equals (1-Q), and TFR is the total fertility rate [20].

### Ethics approval

This study was approved by the Ethical Review Committee for Health Research of the Southern Nations Nationalities and the Peoples' Regional State (SNNPRS) Health Bureau in Ethiopia, and the Regional Committee for Medical and Health Research Ethics of North Norway (REK Nord). We obtained informed oral consent from all of the respondents.

## Results

We interviewed 8870 people of the 9000 sample (98.5% response rate), and included 96% (8503/ 8870) of respondents in the analysis. The missing information from the excluded 4% (367 people) of the respondents was mainly because of misclassification of age (outside the 15–49 year age range) and missing information regarding the gender of the respondents. There were no maternal deaths reported by those excluded from the analysis.

**Figure 1:**
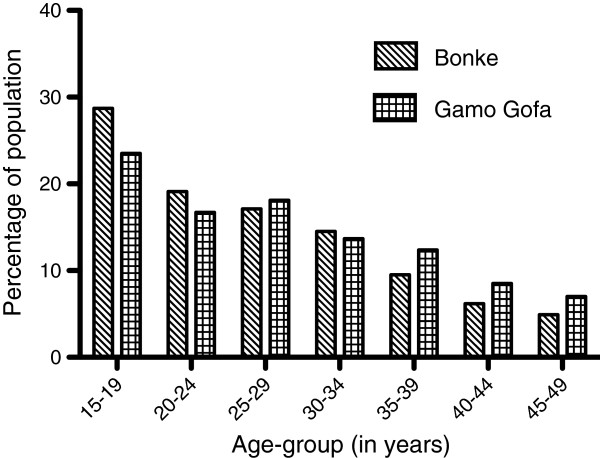
Age distribution of men and women respondents for Bonke sisterhood study 2011 versus Gamo Gofa zone population of the same age group in 2007 Ethiopian National Census.

Of the 8503 respondents in the analysis, 5262 (62%) were men and 3241 (38%) were women. The mean age of the respondents was 26.4 (SD = 8.7) years (range, 15–49 years). The most frequently reported age of the respondents was 30 years, followed by 20 and 18 years (Figure 1).

The 8503 respondents reported 22,473 sisters born to the same mother who survived to the reproductive age. The average number of adult sisters per respondent was 2.6. Of the 22,473 sisters who survived to reproductive age, 2,552 (11.35%) had died. Among the sisters who had died, 32% (819/2552) were pregnancy-related deaths.

The lifetime risk of death from maternal causes was 0.102 (95% CI, 0.096-0.108) or 10.2% (Table 1). Using a TFR of 6.4 for south Ethiopia, we calculated a MMR of 1667 (95% CI, 1564–1769) per 100,000 live births for 1998.

**Table 1:** Maternal mortality estimate using the sisterhood method for the reference period 1998 in rural Bonke, Gamo Gofa, south-west Ethiopia, 2011

**Age of respondents**	**No. of respondents**	**sisters survived age ≥ 15 yrs**	**Dead from all causes**	**Maternal deaths (r)**	**adjustment factor ( f)**	**Sisters units exposed to risk (β)**	**Lifetime risk (Q )**
	**k**	**e**	**C**	**r**	**f**	**β = ef**	**Q=r/ β**
15-19	2443	6471*	428	240	0.107	693	0.346
20-24	1625	4306*	370	152	0.206	887	0.172
25-29	1450	3889	375	152	0.343	1334	0.114
30-34	1235	3135	358	103	0.503	1576	0.065
35-39	812	2201	331	89	0.664	1461	0.061
40-44	523	1397	255	52	0.802	1120	0.046
45-49	415	1074	225	31	0.900	997	0.031
Total	8503	22473	2342	819		8068	0.102

*inflated number of sisters obtained by multiplying the average number of sisters survived for respondents aged 25–49 (which is 2.65 in this data) by the number of respondents in the younger age groups (age 15–19 and 20–24). Originally reported sisters by the young group were: 5425 for aged 15–19 and 4230 for 20–24 years old respondents.

Table 2 also shows estimates obtained from male and female sibling respondents separately. The lifetime risk estimate based on male respondents was 0.095 (95% CI, 0.086-0.105) with a corresponding MMR of 1547 (95% CI,1395-1718) per 100,000 live births (LB). A similar estimate based on information from female respondents provided a slightly higher lifetime risk of 0.121 (95% CI, 0.104-0.127) and MMR of 1995 (95% CI, 1701–2099) per 100,000 LB.

**Table 2:** Maternal mortality indicators estimated separately for male and female respondents using the sisterhood method for year 1998 in rural Bonke, Gamo Gofa, South-west Ethiopia, 2011

	**Male**	**Female**	**total**
Number of respondents	5262 (62%)	3241(38%)	8503
Sisters survived 15 years+	11235	8838	22473
Sisters died of all causes	1483	859	2342
Pregnancy related deaths	482	337	819
Sister units of risk exposure	5094	2785	8068
Lifetime risk of maternal death	0.095	0.121	0.102
MMR*	1,547	1,995	1,667

* per 100,000 live births.

## Discussion

We calculated a lifetime risk of maternal mortality of 10.2%, which corresponded to a MMR of 1667 per 100,000 LB in 1998. There have been no prior community-based maternal mortality estimations from Gamo Gofa, and our study presents the highest estimate for community-based studies using the sisterhood method in Ethiopia.

In Butajira, which is in south central Ethiopia, the MMR was estimated to be 665 per 100, 000 LB in 1996 using the sisterhood method [11]. The Butajira study might have been methodologically more robust than the current study as it was linked to demographic surveillance and probably had a more precise age estimation. However, Butajira also had better access to health services, and this could also explain the differences in MMR compared with Bonke. Shiferaw et al. [10] reported a MMR of 570 per 100,000 LB from Illubabor in western Ethiopia in 1991; however, both studies reported MMR rates below the international estimates for Ethiopia at that time.

Hill and colleagues [3] estimated the MMR for Ethiopia in 1995 to be 1814 per 100,000 LB, which was similar to our finding. Our estimate was close to the natural MMR expected without access to contemporary obstetric care. We believe the impoverished and rural Bonke area had a high MMR in recent decades when the population had no access to basic and comprehensive emergency obstetric care because the population resided in isolated villages with limited transportation. A recent national survey in 2008 showed that 7% of all deliveries take place with health care facilities, and only 3% of facilities provide comprehensive emergency obstetric care [22]. Taking in to account the year of the estimate (1998) and the typical rural location of Bonke, our estimate may have reflected the reality the Bonke women experienced. Also, between 1996 and 2000 there were severe malaria epidemics in southern Ethiopia, and the Bonke lowlands was no exception, which might have caused additional maternal deaths. High MMRs have been associated with high HIV prevalence rates elsewhere [23]. In Ethiopia, however, the effect of the HIV epidemic might not have been important as the HIV prevalence was < 1% in rural areas [24].

An alternative explanation for the high MMR in the current study may be that the sisterhood method provides a biased estimate through selection or information errors and data adjustments. With respect to selection bias, we could have obtained information from many siblings on a death that involved a single woman. Such multiple counting is considered the basis for over-estimation. Potential information biases include misreporting of age or recall errors on the timing of maternal deaths, or even non-recognition of early pregnancy-related deaths.

To ensure correct age determination, we asked several probing questions, such as the number of children the respondents had, the year of marriage, and past events (local calendar) to determine the respondent's age. Because Ethiopia has no system of birth registry, determination of age data is uncertain, which could lead to errors, such as digit preference, as observed in our data. Some respondents may also claim to be younger than their real age, as suggested in Figure 1.

With respect to multiple counting as a potential basis for overestimation, Graham et al. [17] argued that because the sisterhood method is based on a proportional relationship, multiple counting in the denominator is offset by counting sister deaths in the numerator, thus there is no biased result. Trusell et al. [25] emphasized multiple counting of siblings who fall in the sample as essential for the success of the sisterhood method. Therefore, because we did not restrict the siblings during data collection and analysis, we cannot rule out multiple counting, but we believe this is not a major source of bias influencing our estimates.

People often forget past dates of events when responding to research questions. We asked the respondents to recall and report the time and cause of maternal deaths of their adult sisters. Two potential forms of error are of concern. First, the respondent could forget the exact time of the death. This could incorrectly increase the MMR if the respondents reported that the sisters died 6 weeks after pregnancy termination. Second, underreporting could occur if the cause of death was misclassified without recognizing early-pregnancy-and abortion-related deaths. However, in rural areas there are strong social ties, and events such as pregnancy are announced early, suggesting a reduced risk of missed early pregnancy-related maternal deaths. We attempted to probe respondents, especially those reporting maternal deaths, to ensure the death was within 6 weeks after the pregnancy was terminated. The information provided is most likely accurate because the 6-week period is the time that most mothers remain at home. The Gachino tradition of women staying at home after delivery is strictly followed by the rural Bonke population.

The 95% CI of our MMR estimate was narrower compared to some other reports using similar methods. The interval was calculated from the 95% CI of the lifetime risk, which in turn depends on the number of maternal deaths counted in the study. In the current study we expected a minimum number of maternal deaths of 384 for 8 000 respondents; however, there was actually 8503 respondents and 819 reported maternal deaths. The large number of maternal deaths that resulted in the narrow 95% CI of the lifetime risk may have caused a narrow interval in the MMR.

We also showed separate estimates based on information from male and female respondents (Table 2). In the current study there were more male respondents than women respondents, which may have occurred for the following reasons: women usually travel to rural open market places that are often far from home in Bonke; and in rural Ethiopia women usually sit in a hidden part of their home ('Guada' in Amharic) as men chat in the living room, thus women may not be available for interview as they shy away from interviewers. Nevertheless, the estimates were similar with a slight increase for female respondents, which could be because of the close relationship sisters have with each other regarding the sharing of information, such as pregnancy. Most previous studies have only enrolled female respondents, despite the recommendation by Graham and colleagues [17] in their original introduction of the sisterhood method to include men in subsequent studies. Male respondents can more easily be accessed for interviews in rural places where they gather for social meetings than women who mostly stay at home or travel to market places. Thus, in future studies interviewing men alone may be an efficient way to reduce the house-to-house visits in search of women respondents among the often scattered households in rural areas.

Although the estimate obtained by the sisterhood method cannot be used to make geographic comparisons and time trend changes, it is useful in providing the magnitude of the situation in a given area. There have been several policy interventions implemented by the Ethiopian government during the past decade. We consider it encouraging that the public health authorities are using Emergency Obstetric Care Guidelines for improving health care in resource-poor settings, and are working to strengthen the referral system. The Ethiopian Government is also setting up primary hospitals for every 100,000 population, and thus improving access to health care. Other important interventions include a malaria prevention campaign through the distribution of bed nets for households, the introduction of two health extension works to all rural villages, training and posting of midwives and health offices, and rapid expansion of health centres. Therefore, the findings of this study may help establish a baseline to assess the current situation and the effects of the interventions using other methods, such as household surveys.

## Conclusion

Our findings suggest that people living in remote and underprivileged Bonke have high MMRs. This highlights the importance to strengthen lifesaving comprehensive emergency obstetric care in this area, and in similar rural areas in Ethiopia. Because of uncertainties in our estimates, we also advise using alternative sources of information, such as birth registries and short recall-period household interviews to improve the accuracy of the MMR estimate.

## Competing interests

We declare we have no competing interests. YY receives a PhD stipend from The Norwegian state loan for higher education, and BL receives a salary from the University of Bergen in Norway.

## Authors’ contributions

YY designed the study, organized the data collection, analyzed the data, and wrote the first draft of the manuscript. BL participated in the design of the study, supervised the entire process, and reviewed and modified the drafts of the manuscript. Both authors revised and approved the final draft of the manuscript.

## Pre-publication history

The pre-publication history for this paper can be accessed here:

http://www.biomedcentral.com/1471-2393/12/136/prepub
